# Supplementary material for: Gender diversity and profit efficiency of microfinance institutions: A Sub-Saharan study
Source: PLoS One. 2024 Aug 5;19(8):e0307758. doi: 10.1371/journal.pone.0307758 (PMC11299832; doi:10.1371/journal.pone.0307758)
Supplement: Attachment — Submitted filename: REVIEW REPORT-PLOS 2024.pdf [file pone.0307758.s002.pdf]

Title of manuscript: Gender diversity and profit efficiency of microfinance institutions: A Sub-Saharan study

GENERAL COMMENTS

The abstract of the study which I reproduce below sums up the focus of the study:

Irrespective of the promising opportunity to improve profit efficiency by at least 73%,

microfinance institutions operating in Sub-Saharan Africa are efficient only for 27%, far below the average value. The conclusion is drawn after analyzing the profit efficiency of the microfinance institutions using the stochastic frontier approach applied to data obtained from 128 microfinance institutions operating in 34 Sub-Saharan African countries. The study results suggest the existence of uniform profit efficiency experience from time to time due to the lack of sufficient effort exerted to improve efficiency concurrent with the changes in technology and techniques of financial services provision. Microfinance institutions operating in low-income countries and credit union form microfinance are economically more efficient than their counterparts. Furthermore, the profit efficiency of microfinance institutions is significantly affected by total assets, cost per loan, loan per staff, legal status, and the county's income group of the microfinance. Notably, the profit efficiency of microfinance institutions is adversely affected by the presence of female borrowers and female loan officers suggests that gender diversity plays a role in efficiency of microfinance institutions. Finally, we recommend that the managing body of microfinance work more on improving labor efficiency, earning asset utilization, loan collection efficiency, women's involvement and the hottest technology implementation.

The manuscript is on an interesting topic. The findings are insightful. However, its current form cannot be published until and unless the concerns below are addressed:

ABSTRACT: In the abstract of the manuscript authors have made this recommendation: "***Finally, we recommend that the managing body of microfinance work more on improving labor efficiency, earning asset utilization, loan collection efficiency, women's involvement and the hottest technology implementation."*** Please, on what findings have this recommendation been made? For instance, on what basis are authors have authors recommended more women involvement when they have stated in the second paragraph of the "Conclusions and Managerial Implications section" of their manuscript that "regarding gender diversity, the profit efficiency of microfinance institutions is adversely affected by the presence of female borrowers and female loan officers"? The recommendations of a study must be based on its findings. They should not spring from conjectures and imaginations!

Indeed, authors must rewrite the entire abstract to make it more concise devoid of unnecessary embellishment!

INTRODUCTION: Authors have not made a good case for their study. What gap(s) have authors attempted to fill or bridge? Accordingly, they are invited to improve the introduction to the manuscript by making a cogent case for it.

CONTRIBUTION OF THE STUDY: Contribution of the study should be rewritten. Authors must demonstrate the relevance of their study to the extant literature in terms of knowledge, managerial practice and theory. Authors have attempted to identify the contributions of their manuscript but what they have done is not convincing at all. Besides, authors have made inconsistent claims in the number of ways in which their study expands the frontiers of the existing knowledge.

"This study contributes to the existing stock of knowledge in three main ways" This appears in the last but one paragraph of the introduction.

"Moreover, the study findings contribute to the literature in two other ways." This appears in the last paragraph of the theoretical and empirical review section of the paper.

Authors should clarify the number of ways the study in which their study contributes to the extant literature.

LITERATURE REVIEW: Authors have a dedicated section in their paper captioned "Theoretical and Empirical Review". Under this section they have reviewed three theories: resource dependence theory, agency theory and social identity theory. However, they have failed to use these theories to explain or predict the relationship between the variables of interest in their study: Gender diversity and profit efficiency. This is fatal.

The empirical review part is too terse. Authors must improve it to make it possible for the reader to locate their findings in the empirical literature.

CONCLUSIONS AND MANAGERIAL IMPLICATIONS: This side of the manuscript requires a major improvement. What authors have written is nothing more than the rehash of the findings of the study. Besides, some vague statements have been made by authors. For example, authors have stated: "A uniform profit efficiency level was recognized by the MFIs in SSA from time to time due to the absence of self-advancement and management in line with the rapid changes in technology and technique." What is the meaning of the above statement? Authors are advised to spend some time to draw inferences from their results as well as delineate the managerial implications of their findings.

LANGUAGE: “have been presented their annual financial report to the global microfinance information exchange” This should be corrected. I think “been” is not needed in the above phrase which appears in the first sentence under Sampling and Data source sub-section of the Material and Methods Section of the manuscript. Authors are advised to stick with one tense. They have mixed tenses. They should either write in the simple present tense or past tense. They should not mix tenses.

RECOMMENDATION: The manuscript is on an interesting topic that has the potential to make a valuable contribution to the extant literature. However, its current form cannot be published. It requires some significant improvement to bring it to the level suitable for publication. I have pointed out my main issues of concern in this report. Consequently, I recommend a major revision.
