# Supplementary material for: Gender diversity and profit efficiency of microfinance institutions: A Sub-Saharan study
Source: PLoS One. 2024 Aug 5;19(8):e0307758. doi: 10.1371/journal.pone.0307758 (PMC11299832; doi:10.1371/journal.pone.0307758)
Supplement: Attachment — Submitted filename: Response to reviewers 1 (letter).pdf [file pone.0307758.s003.pdf]

Date: February 07, 2024

To: Academic Editor and Reviewer of PLOS ONE

Subject: Reponses to reviewer #1 comments

Hello dear all, I thank you very much for your time, constructive comments and supports regarding our manuscript titled "Gender diversity and profit efficiency of MFIs: A Sub-Saharan Africa study."

Manuscript code: PONE – D – 23 – 42063 – EMID: 452ead2fc03182eb

Comment 1: **ABSTRACT**

- "Indeed, authors must rewrite the entire abstract to make it more concise devoid of unnecessary embellishment."

Response 1: following your informative comment regarding the abstract of our study, we have modified the recommendation section (heighted in yellow) of the abstract based on the direct findings of the study. Dear reviewer, the rewritten abstract is included in the manuscript as it is presented below.

Abstract

Irrespective of the promising opportunity to improve profit efficiency by at least 73%, microfinance institutions operating in Sub-Saharan Africa are efficient only for 27%, far below the average value. The conclusion is drawn after analyzing the profit efficiency of the microfinance institutions using the stochastic frontier approach applied to data obtained from 128 microfinance institutions operating in 34 Sub-Saharan African countries. The study results suggest the existence of uniform profit efficiency experience from time to time due to the lack of sufficient effort exerted to improve efficiency concurrent with the changes in technology and techniques of financial services provision. Microfinance institutions operating in low-income countries and credit union form microfinance are economically more efficient than their counterparts. Furthermore, the profit efficiency of microfinance institutions is significantly affected by total assets, cost per loan, loan per staff, legal status, and the county's income group of the microfinance. Notably, the profit efficiency of microfinance institutions is adversely affected by the presence of female borrowers and female loan officers suggests that gender diversity plays a role in efficiency of microfinance institutions. Finally, we recommend that the managing body of microfinance institutions to work more on improving labor efficiency, earning asset utilization, loan collection efficiency, and implement self-improvement strategies to enhance the profit efficiency of the institutions.

Comment 2: **INTRODUCTION:**

- "Authors have not made a good case for their study. What gap(s) have authors attempted to fill or bridge? Accordingly, they are invited to improve the introduction to the manuscript by making a cogent case for it."

Response 2: Dear reviewer, following your constructive comments, we have tried to show the gaps of exiting literature and the contribution of our study in introduction section of the revised manuscript. The following paragraph is taken from the introduction section to show the need for study and tangible problems of MFIs that attract attention of scholars like us. The highlighted statement is added to the paragraph to describe statement of the problem in better way.

…… The stakeholders also argue for dual missions of MFIs in their operations. In the social mission, MFIs are designed to provide cheaper financial services to low-income people, women, and those excluded from mainstream financial institutions. In the economic mission, they are expected to generate profit from their operations to maintain a sustainable and stable financial position [9, 18]. However, the efficiency of MFIs is a prerequisite to achieve the missions [1]. Improving efficiency by working on profit maximization is an expressway for MFIs to reach their goals. Hence, scholars working inside and outside Africa are urging MFIs operating on the continent to focus on improving their technical efficiency [13], labor productivity [1, 35], wise utilization of resources and increasing output [31]. These opinions imply the institutions are economically inefficient in their operations in general and profitability in particular. Likewise, [1] suggested that the efficiency of MFIs in Africa is less than 41% implying the presence of wide space for further improvement in efficiency, by approximately 60% on average.

Comment 3: **CONTRIBUTION OF THE STUDY**

- “Contribution of the study should be rewritten and authors should clarify the number of ways the study in which their study contributes to the extant literature.”

Response 3: Dear reviewer, thank you very much for your in-depth and insightful comments. Regarding the contribution of the study, we have tried to see the effect of gender diversity from five perspectives – board member, management member, loan officer, regular personnel and borrower, which were not addressed by the exiting literature. Previous empirical studies were give emphases for gender diversity aspects from two perspectives such as board member and borrower gender diversity. However, as reports of the World Bank show (MIX, 2021), women may engage on at least three additional roles in MFIs. Namely, management member, loan officer, regular personnel, thus, we could extend the existing knowledge by adding three roles of women in MFIs to our study design.

Regarding the number of contributions, the inconsistency is now corrected in the last paragraph under theoretical and literature review section and the corrected paragraph (highlighted in yellow) are presented as follows.

…. This study aims to assess the association between women’s roles and the profit efficiency of MFIs in SSA from five aspects of gender diversity. Moreover, the generalizability of the study findings is improved for taking a large number of MFIs operating in all SSA countries for a longer period (2009 – 2018), and for applying a parametric panel data analysis technique, i.e.,

the stochastic frontier approach (SFA), unlike those studies adopted nonparametric data analysis approaches in the MFI literature.

Comment 4: **LITERATURE REVIEW**

- “However, they have failed to use these theories to explain or predict the relationship between the variables of interest in their study: Gender diversity and profit efficiency.”

Response 4: Dear reviewer, we have tried to bridge the postulate of existing theories with the view of our study findings using the statements presented in the following paragraph (highlighted in yellow). The statement exists in the revised manuscript. We hope these statements well explained the relationship between gender diversity and profit efficiency.

… Theoretically, these empirical findings present notable remarks. The adverse effects of borrowers’ and loan officers’ gender diversity against the profit efficiency of MFIs contradict the concepts of resource dependency theory, resource-based view and agency theory. However, the findings are consistent with the concept of social identity theory. It seems that the institutions serve women in borrowing services and allow them to hold officer positions, probably for the desire to meet regulation requirements for gender diversity at work. As a result, women receiving borrowing services from the institutions and working in the institutions may face group isolation and ignorance by their counterparts, and the institutions are limited in effectively extracting and using the inherent potential of women in the workplace.

Comment 5: **CONCLUSIONS AND MANAGERIAL IMPLICATIONS**

- “This side of the manuscript requires a major improvement. What authors have written is nothing more than the rehash of the findings of the study. Besides, some vague statements have been made by authors. For example, authors have stated: “A uniform profit efficiency level was recognized by the MFIs in SSA from time to time due to the absence of self-advancement and management in line with the rapid changes in technology and technique.” What is the meaning of the above statement? Authors are advised to spend some time to draw inferences from their results as well as delineate the managerial implications of their findings.”

Response 5: Following your constructive comment in this section, we have restated the previous conclusions and managerial implications in more informative ways as presented in the following passage (highlighted in yellow) and the main manuscript is rewritten accordingly.

… MFIs operating in SSA realize profit efficiency below the average, only 27%. Credit union MFIs and MFIs operating in low-income countries are more efficient in realizing profit than other forms of MFIs and institutions operating in the upper-middle-income group. A uniform profit efficiency level was recognized by the MFIs over the study period that shows the absence of self-improvement in operations, interest expense management, human resource management and efficient utilization of earning assets in line with the rapid changes in financial services provision technology and techniques. Input prices such as the labor price and price of a fund and the output item, particularly other earning assets, play significant roles in the profit efficiency of MFIs operating in SSA.

Moreover, firm size, cost per loan, loan per personnel, legal status, and country's income group significantly affect the profit efficiency of MFIs in SSA. Regarding gender diversity, the profit efficiency of microfinance institutions is adversely affected by the presence of female borrowers and female loan officers. It is necessary to work more on improving profit efficiency by designing alternative policy and updating existing strategies that could enhance labor productivity, reduce loan-related costs, capacitate women borrowers and increase loan collection efficiency.

To improve profit efficiency, MFIs in SAA are expected to undertake the following managerial actions and decisions. First, capacity-building reforms and training should be implemented to enhance labor productivity, ensure effective utilization of assets and reduce loan-operating costs. Second, redesign human resources policy and loan provision regulations to ensure women's involvement on the board member, management, personnel and borrowing services that enable the MFIs to take gender diversity advantages in making sound decisions, resource provisions and cost management. Third, focus on removing work environment practices that expose women to self-categorization and isolation, and deteriorating their confidence to take full responsibility in decision-making. Generally, in the desire to improve profit efficiency, MFIs in SSA should reduce holding of the less productive labor force, minimize costly practices of producing loans, and make women empowerment and capacity building the core part of financial services provision.

Comment 6: **LANGUAGE**

- "…have been presented their annual financial report to the global microfinance information exchange" This should be corrected. I think "been" is not needed in the above phrase which appears in the first sentence under Sampling and Data source sub-section of the Material and Methods Section of the manuscript. Authors are advised to stick with one tense. They have mixed tenses. They should either write in the simple present tense or past tense. They should not mix tenses."

Response 6: Dear reviewer, the statement is corrected by removing "been" as … "have presented their annual financial report to the global microfinance information exchange …" in the revised manuscript.

****** Thank you very much*******

Kind regards

Tarekegn Tariku Ebissa

The corresponding author
