# Supplementary material for: Gender diversity and profit efficiency of microfinance institutions: A Sub-Saharan study
Source: PLoS One. 2024 Aug 5;19(8):e0307758. doi: 10.1371/journal.pone.0307758 (PMC11299832; doi:10.1371/journal.pone.0307758)
Supplement: Attachment — Submitted filename: R2 response letter to editor & reviewer 3.docx [file pone.0307758.s004.docx]

Date: June 29, 2024

To: Editor and Reviewer **#3** of PLOS ONE

**Subject:** **Reponses to reviewer #3 and editor comments**

Hello dear all, I thank you very much for your time, constructive comments and supports regarding our manuscript titled “Gender diversity and profit efficiency of MFIs: A Sub-Saharan Africa study.”

Revision code: PONE-D-23-42063R1

Manuscript code: PONE – D – 23 – 42063 – EMID: 452ead2fc03182eb

**Reviewer #3 comments**

1. **Abstract**

- "The abstract is too revealing. It should be as concise as possible”

Authors’ response

Dear reviewer, earlier abstract is revised following your constructive comments by removing long statement and paraphrasing sentences. The revised version of the abstract is presented below and changes are highlighted in yellow.

“*Irrespective of the promising opportunity to improve profit efficiency by at least 73%, microfinance institutions operating in Sub-Saharan Africa are efficient only for 27%, far below the average value. The conclusion is drawn after analyzing the profit efficiency of the microfinance institutions using the stochastic frontier approach applied to data obtained from 128 microfinance institutions operating in 34 Sub-Saharan African countries. The study results suggest the presence of uniform profit efficiency experience across time among microfinance institutions. Microfinance institutions operating in low-income countries and credit union form microfinance are economically more efficient than their counterparts. Furthermore, the profit efficiency of microfinance institutions is significantly affected by total assets, cost per loan, loan per staff, legal status, and the county’s income group of microfinance. Notably, the profit efficiency of microfinance institutions is adversely affected by the presence of female borrowers and female loan officers suggesting that gender diversity plays a role in the efficiency of microfinance institutions. Finally, we recommend that the managing body of microfinance work more on improving labor efficiency, earning asset utilization, loan collection efficiency, women’s involvement and the hottest technology implementation*.”

2. **Introduction**

“The introduction can be better written”

Authors’ response

Dear reviewer, the introduction sections are rewritten by removing the some confusing phrases and statement. The revised introduction sections for paragraph one and two are presented below with heighted mark and included in the revised manuscript as well.

“Microfinance institutions (MFIs) are vibrant tools for fighting poverty by reaching low-income people and disadvantaged groups such as women by creating affordable and easily accessible financial services. In addition, MFIs play remarkable roles in economic growth by providing services to rural people, reducing transaction costs, easing loan requirements (collateral), smoothing consumption, ensuring gender equality, and lending to small-scale borrowers [4, 9, 24, 29]. The roles are more helpful and fruitful in the SSA region than in other parts of the world since the region is occupied by the least banked households and the highest number of low-income people [19]. The good news is that approximately 917 MFIs are currently operating in the SSA region [26].

Furthermore, MFIs have dual missions to achieve in their operations. In the social mission, MFIs are designed to provide cheaper financial services to low-income people, women, and those excluded from mainstream financial institutions. In the economic mission, they are expected to generate profit from their operations to maintain a sustainable and stable financial position [9, 18]. However, the efficiency of MFIs is a prerequisite to achieving these missions [1]. Improving efficiency by working on profit maximization is an expressway for MFIs to reach their goals. Hence, scholars working inside and outside Africa are urging MFIs operating on the continent to focus on improving their technical efficiency [13], labor productivity [1, 35], wise utilization of resources and increasing output [31].”

3. **Motivation of the study and research gap**

Authors’ response

Dear reviewer, in this revision we have presented additional empirical literature gaps by critiquing the limitations of recent studies and showing the contributions (motivations) of our study. The revised section is presented below and the changes are highlighted.

-------- Despite the above facts, very few studies have been conducted to investigate the profit efficiency of MFIs operating in SSA, and the empirical gaps are remarkable. Empirical studies in the literature are limited in applying stochastic frontier analysis (SFA) – the parametric approach – [3, 9, 31], covering a large number of MFIs [29] and assessing the roles of gender diversity in the profit efficiency of MFIs. For instance, a recent study assessed the effect of women’s participation on the cost efficiency of MFIs in Sub-Sahara Africa fails to address the profit efficiency aspects of the institutions [40]. Unlike our study, the empirical works of [41, 42, 43, 44] focused on addressing the effects of gender diversity on financial performance, social performance and technical efficiency of MFIs in Sub-Saharan Africa. Rather our study emphasized the effects of gender diversity on the profit efficiency of the institutions.

4. **Conclusions of the study**

Authors’ response

Dear reviewer, we tried to draw the conclusions of our study from the findings of the study and we could have a meaningful recommendation and managerial implications for MFIs operating in the study area. The revised version of conclusion section is presented below with highlighted marks.

“MFIs operating in SSA realize profit efficiency below the average, only 27%. It indicates the presence of potential opportunities to raise the current profitability position of MFIs in SSA by improving their efficiency level by 73%. Credit union MFIs and MFIs operating in low-income countries are more efficient in realizing profit efficiency than other forms of MFIs and institutions operating in the upper-middle-income group. A uniform profit efficiency level was recognized by the MFIs in SSA from time to time due to the absence of self-advancement and management in line with the rapid changes in technology, and financial services provision techniques. Input prices such as the labor price and price of a fund and the output item, particularly other earning assets, play significant roles in the profit efficiency of MFIs operating in SSA.”

**Response to editor’s comment and journal requirement**

Dear editor, we have cited five new empirical studies in the body of the text and included in the reference section in response to the reviewer comments to revise empirical research gaps and clarify motivation of our study. In addition, the presence of all in text citation and inclusion in the reference are verified rigorously.

Dear editor and reviewer, we thank you very much.

Kind regards

Tarekegn Tariku Ebissa

The corresponding author

**Note: table of correction is attached with this response letter in the next pages for your quick crosscheck.**

**Table of correction**

| **S.No** | **Revised section** | **Revised content (changes are highlighted in yellow)** | **Page** |
| --- | --- | --- | --- |
| 1 | Abstract | “*Irrespective of the promising opportunity to improve profit efficiency by at least 73%, microfinance institutions operating in Sub-Saharan Africa are efficient only for 27%, far below the average value. The conclusion is drawn after analyzing the profit efficiency of the microfinance institutions using the stochastic frontier approach applied to data obtained from 128 microfinance institutions operating in 34 Sub-Saharan African countries. The study results suggest the presence of uniform profit efficiency experience across time among microfinance institutions. Microfinance institutions operating in low-income countries and credit union form microfinance are economically more efficient than their counterparts. Furthermore, the profit efficiency of microfinance institutions is significantly affected by total assets, cost per loan, loan per staff, legal status, and the county’s income group of microfinance. Notably, the profit efficiency of microfinance institutions is adversely affected by the presence of female borrowers and female loan officers suggesting that gender diversity plays a role in the efficiency of microfinance institutions. Finally, we recommend that the managing body of microfinance work more on improving labor efficiency, earning asset utilization, loan collection efficiency, women’s involvement and the hottest technology implementation*.” | 2 |
| 2 | Introduction | “Microfinance institutions (MFIs) are vibrant tools for fighting poverty by reaching low-income people and disadvantaged groups such as women by creating affordable and easily accessible financial services. In addition, MFIs play remarkable roles in economic growth by providing services to rural people, reducing transaction costs, easing loan requirements (collateral), smoothing consumption, ensuring gender equality, and lending to small-scale borrowers [4, 9, 24, 29]. The roles are more helpful and fruitful in the SSA region than in other parts of the world since the region is occupied by the least banked households and the highest number of low-income people [19]. The good news is that approximately 917 MFIs are currently operating in the SSA region [26].  Furthermore, MFIs have dual missions to achieve in their operations. In the social mission, MFIs are designed to provide cheaper financial services to low-income people, women, and those excluded from mainstream financial institutions. In the economic mission, they are expected to generate profit from their operations to maintain a sustainable and stable financial position [9, 18]. However, the efficiency of MFIs is a prerequisite to achieving these missions [1]. Improving efficiency by working on profit maximization is an expressway for MFIs to reach their goals. Hence, scholars working inside and outside Africa are urging MFIs operating on the continent to focus on improving their technical efficiency [13], labor productivity [1, 35], wise utilization of resources and increasing output [31].” | 2 |
| 3 | Motivation of the study and research gap | Despite the above facts, very few studies have been conducted to investigate the profit efficiency of MFIs operating in SSA, and the empirical gaps are remarkable. Empirical studies in the literature are limited in applying stochastic frontier analysis (SFA) – the parametric approach – [3, 9, 31], covering a large number of MFIs [29] and assessing the roles of gender diversity in the profit efficiency of MFIs. For instance, a recent study assessed the effect of women’s participation on the cost efficiency of MFIs in Sub-Sahara Africa fails to address the profit efficiency aspects of the institutions [40]. Unlike our study, the empirical works of [41, 42, 43, 44] focused on addressing the effects of gender diversity on financial performance, social performance and technical efficiency of MFIs in Sub-Saharan Africa. Rather our study emphasized the effects of gender diversity on the profit efficiency of the institutions. | 3 |
| 4 | Conclusion | MFIs operating in SSA realize profit efficiency below the average, only 27%. It indicates the presence of potential opportunities to raise the current profitability position of MFIs in SSA by improving their efficiency level by 73%. Credit union MFIs and MFIs operating in low-income countries are more efficient in realizing profit efficiency than other forms of MFIs and institutions operating in the upper-middle-income group. A uniform profit efficiency level was recognized by the MFIs in SSA from time to time due to the absence of self-advancement and management in line with the rapid changes in technology, and financial services provision techniques. Input prices such as the labor price and price of a fund and the output item, particularly other earning assets, play significant roles in the profit efficiency of MFIs operating in SSA. | 18 |
